# Seven year experience with a mandatory training course within the Vigigerme® infection control program

**DOI:** 10.1186/1753-6561-5-S6-P278

**Published:** 2011-06-29

**Authors:** J Pasricha, S Longet-di Pietro, H Sax

**Affiliations:** 1 infection control, University of Geneva Hospital, Geneva, Switzerland

## Introduction / objectives

VigiGerme^®^ is an infection control program built on social marketing principles at the University of Geneva Hospital (HUG). The 2-hour training course was introduced in 2003 and has been mandatory for all healthcare workers (HCWs) since.

## Methods

All participants completed a 23-item questionnaire assessing their attitudes (A) towards and knowledge (K) of infection control at the onset and end of the course, and a course evaluation. We performed a descriptive analysis of all questionnaires from 2003 to 2010. Likert scales (1-7) were transformed to binomial values (1-5=0; >6=1).

## Results

Of 10373 participants, complete responses were obtained from 9455 (91.4%): 42% nurses, 20% physicians, 26% nursing assistants, and 12% other professional categories. The proportion of participants providing correct responses to questions on hand hygiene, glove use, and mask use increased after the course from 86-97%, 69-93%, and 58-77%, respectively (all p<.001). Perceived high institutional safety culture (SC) and HCW accountability for infectious outcomes (ACC) changed from 20-57% and 55-87% respectively. Furthermore, there was a shift in baseline (pre-course) SC and ACC responses over time (2003-10) from16 to 35% and from 50 to 72%, respectively (all P<.001). Clinical scenarios testing knowledge of isolation precautions (IP) increased from 0.5 to 10.4% (aggregate of 4 correct responses; p<.001). Course evaluation averaged at 6 of 7 points.

## Conclusion

The course is highly appreciated by HCW and influences attitude and knowledge positively. Identification of correct IP remains challenging for HCWs. Interestingly, there was a spontaneous evolution in perception of safety culture and HCW accountability over the 7 years.

## Disclosure of interest

None declared.

